# Using standardized patients to assess communication skills in medical and nursing Students

**DOI:** 10.1186/1472-6920-10-24

**Published:** 2010-03-17

**Authors:** C Anthony Ryan, Nuala Walshe, Robert Gaffney, Andrew Shanks, Louise Burgoyne, Connie M Wiskin

**Affiliations:** 1 School of Medicine, College of Medicine and Health, Brookfield Health Sciences Complex, University College Cork (UCC), Ireland; 2 School of Nursing, College of Medicine and Health, Brookfield Health Sciences Complex, University College Cork (UCC), Ireland; 3 Integrative Learning Centre, University of Birmingham, Birmingham, UK

## Abstract

**Background:**

A number of recent developments in medical and nursing education have highlighted the importance of communication and consultation skills (CCS). Although such skills are taught in all medical and nursing undergraduate curriculums, there is no comprehensive screening or assessment programme of CCS using professionally trained Standardized Patients Educators (SPE's) in Ireland. This study was designed to test the content, process and acceptability of a screening programme in CCS with Irish medical and nursing students using trained SPE's and a previously validated global rating scale for CCS.

**Methods:**

Eight tutors from the Schools of Nursing and Medicine at University College Cork were trained in the use of a validated communication skills and attitudes holistic assessment tool. A total of forty six medical students (Year 2 of 5) and sixty four nursing students (Year 2/3 of 4) were selected to under go individual CCS assessment by the tutors via an SPE led scenario. Immediate formative feedback was provided by the SPE's for the students. Students who did not pass the assessment were referred for remediation CCS learning.

**Results:**

Almost three quarters of medical students (33/46; 72%) and 81% of nursing students (56/64) passed the CCS assessment in both communication and attitudes categories. All nursing students had English as their first language. Nine of thirteen medical students referred for enhanced learning in CCS did not have English as their first language.

**Conclusions:**

A significant proportion of both medical and nursing students required referral for enhanced training in CCS. Medical students requiring enhanced training were more likely not to have English as a first language.

## Background

Modern health care has become extremely complex and it continues to grow in complexity because of economic pressures (new levels of efficiency and productivity), and as a result of the increasing capabilities of modern medicine [1]. In addition, enhanced patient rights, autonomy and expectations entail that while students need access to patients in order to learn, patients views on who examines them and their rights to rest and privacy must be respected [2]. Yet the demand for patient access continues unabated across the wide range of health professionals in training. As an example, the Fottrell Report on the future of medical education in Ireland recommends an increase in the intake of students from a level of 395 per annum with 62% non-EU students in 2003, to 725 per annum with a projected 25% of places targeted for non-EU intake [3]. The above issues and other potent drives of change require innovative, simulated teaching for the novice student, particularly in the non-cognitive areas of communication, interpersonal development and reflective practice.

Health care students may benefit from training through simulation scenarios, prior to experiencing the complexity of "real" patients. Practicing CCS in a mistake-forgiving environment allows students to make, recognize, and correct errors in a non-threatening, trainee-centred environment with immediate formative feedback. Importantly, the same encounters highlight personal strengths and can build student confidence. For near-authenticity, standardized patient educators (SPEs) are used worldwide for teaching and testing CCS in order to prepare students for encounters with authentic patients. The use of SPE's guards against the real patient, a genuinely ill and possibly frightened individual, encountering an inexperienced or inadequate communicator.

In the early 1960's, standardized patients were created by Howard Barrows [4,5]. Barrows noted in his clinical work with students that, in the absence of observation and feedback, errors in practice persisted. He also acknowledged a lack of standardization using real patients in high stakes examination, an observation shared by Harden in development of the OSCE [6]. Barrows developed the first checklist for standardized patients to record systematically what physical maneuvers the trainee did during the encounter [7]. Thus SP's became educators (SPE's).

The SPE has become one of the most prominent evolving methodologies in medical education. Canadian doctors were innovators in introducing SPE's to Medical Schools in the early 1970's. Then, in 1993, they were the first to use SPE's in a "high stakes" licensure examination by the Medical Council of Canada. The Americans adopted SPE's into their Educational Commission for Foreign Medical Graduates examinations in 1998. By then, 79% of American Medical Colleges Medical Schools used SPE's to teach their CCS. Feedback by SPE was used in 76% of schools, and summative assessment by SPE was used in 70% of schools [2]. In the UK in 1993 the GMC demanded communication training in all UK medical curricula (Tomorrow's Doctors), by which time a number of Schools already had comprehensive programmes in place. Although role-playing volunteers are used in clinical skills laboratories, to our knowledge, there is no formal, professional SPE programme in Ireland.

The purpose of this international, interdisciplinary, collaborative study was to evaluate the acceptability of introducing an SPE programme to assess CCS in medical and nursing students at University College Cork (UCC), using SPE's and a validated holistic rating scale for the assessment of CCS. We invited a group of experienced academic raters of CCS and SPE's from Birmingham University to train Irish academics as raters and to train lay volunteers as SPE's. We took the opportunity to assess a cohort of nursing and medical students who had never previously been exposed to SPE's, and who had never been formally assessed in their CCS.

## Methods

The CCS trainers and SPE's came from the Interactive Studies Unit (ISU) at the University of Birmingham which began teaching communication skills to undergraduates in 1991. It is involved in teaching and assessing CCS in the undergraduate programmes in Medicine, Nursing Studies and Dentistry. The ISU has worked extensively with the West Midlands Deanery, but also delivers nationally and internationally. The ISU offers expertise (and SPE's) for postgraduate teaching, recruitment and testing. They run a substantial coaching programme for referred ('poorly performing') doctors' and have produced numbers of audio visual training products. The ISU was conceived from the outset as a multi-disciplinary educational team. The core staff has qualifications in Medicine, Nursing, Education, Linguistics, Health Care Ethics, Literature, Communication and Drama. A team of approximately 65 professionally trained SPE's supply teaching support for the ISU.

### Subjects

A total of forty six medical students (Year 2 of a 5 year programme) and sixty four nursing students (Year 2/3 of a 4 year programme) were selected to under go individual CCS assessment via an SPE led scenario. The medical students had just completed their first CCS module. All nursing students had previously completed the required communication modules in year 1 and 2 of the 4 year undergraduate nursing programme.

### Academic Assessors

Eight academic assessors from the nursing and medical Schools, College of Medicine and Health, Cork, were trained by the experienced UK educators. The Irish faculty had not previously worked with SPE's or the global rating scale. However, all were experienced in teaching CCS in their appropriate discipline. During the one-day workshop, they were taught how to assess communication skills and attitudes, using the global rating scale, through video-taped and live role-playing scenarios.

### Assessment of CCS

A universal negotiated, global rating scale for communication skills and attitudes was used in this project. These scales were developed and validated at the University of Birmingham, UK [8]. Separate grading scales for communication skills and professional attitudes were assessed and are presented below. The scale ranges from Grade A to E with grades below C considered as a fail grade warranting referral for remediation.

#### Communication Skills

##### Grade A

Excellent use of: appropriate questioning styles - including effective information gathering. Appropriate levels of eye contact and body posture. Excellent active listening. Clear understanding of demonstrating empathy, rapport-building and acknowledgement of emotional responses.

##### Grade B

**Ge**nerally appropriate use of question styles. Students will use eye contact and body language appropriately and in an engaged manner. Students will use a variety of questions to gather information. They will listen actively, demonstrate empathy consistently, and handle emotion in the role-play in a generally appropriate manner.

##### Grade C

Although students may occasionally be inconsistent or erratic, they will gather information adequately and attempt different questioning styles. Although they may occasionally interrupt inappropriately, they generally listen well, Eye contact and body language will mostly be appropriate, with occasional inconsistencies. Students will demonstrate some empathy and respond to emotion, but perhaps in a clumsy fashion or limited way

##### Grade D

***Screening Referral/Assessment Fail***: Limited range of question styles, or erratic and confused questions students may not listen well, interrupting and impeding role-player concerns. Eye contact and body language may not be appropriate to the content of the encounter. There may be little or no demonstration of empathy, little or no response to patient emotion, and inadequate reflection. Skills used within the encounter will neither be appropriately contextualized, nor meaningfully demonstrated (for example a warm smile during the breaking of bad news).

##### Grade E

***Screening Referral/Assessment Fail***: Students at this level will have serious deficiencies in their skill set. They will demonstrate few if any of the following skills: eye contact, body language, active listening, demonstrating empathy and responding to patient emotion. The will demonstrate little understanding of the context in which they might be used.

#### Communication Attitudes

##### Grade A

Students at this level are likely to appear highly professional, confident and sincere. Advice, when offered, will be given appropriately and responsibly. Students interacting with the role-player will demonstrate good levels of respect, and show no signs of prejudice. Students will engage responsibly with the screening process.

##### Grade B

Students at Grade B demonstrate professionalism and sincerity, although may occasionally appear uncertain. Advice, if offered, will mostly be appropriate and responsible. Students will be respectful and demonstrate attitudes free of prejudice. Students will engage with the screening process and generally answer questions appropriately.

##### Grade C

Self presentation is adequate. Advice, if offered, may at times be inappropriate or appear slightly irresponsible, but this is more likely due to lack of insight than an intention to mislead the role-player. Role-players will be treated respectfully, although clumsiness from the student may result in the role-player occasionally feeling uncomfortable. Students will take the occasion seriously.

##### Grade D

***Screening Referral/Assessment Fail***: Students at this grade will appear unprofessional and may appear to lack sincerity. They may give the impression of a lack of care or interest in the role-play. Advice, if offered, will likely be inappropriate and/or poorly presented. Student responses may sometimes appear stigmatising.

##### Grade E

***Screening Referral/Assessment Fail***: Students will have serious difficulties presenting a professional manner. Little interest in the scenario, role-player, or assessor. Students are likely to demonstrate an uncaring interpersonal style that could be interpreted as arrogant, prejudicial or stigmatising.

### Scenario process

Five scenarios were selected to reflect the students' level of training in CCS. The role-playing scenarios were focussed on the student's discipline (Medicine or Nursing). Students were informed that on this occasion they were not being tested on knowledge relating to the 'patient's' condition (e.g. Diabetes, Epilepsy, Anaesthesia).

### Scenario Assessment

There were 2 assessors present for every student encounter (the SPE and academic assessor). Newly trained academic assessors were matched with a UK trained experienced SPE. Alternatively, a newly trained Irish SPE was matched to an experienced UK academic rater. The students were allocated 3 minutes to read the scenario briefing and a further 12 minutes to complete the scenario. Upon completion of the scenario, the student was asked to leave the room. The assessor and the SPE documented their grade of student performance independently and then negotiated an agreed grade with the SPE for both attitudes and skills. The negotiated grade was recorded as the student grade. The student was recalled and formative feedback, but not the grade, was delivered by the SPE.

### Consent

The Chair of the Clinical Research Ethics Committee (CREC) of the Cork Teaching Hospitals confirmed that study conformed to all the relevant guidelines and that ethical matters have been dealt with appropriately. An information sheet and a consent form (available on request) were given to all students who were invited to participate in the project. Students were enrolled into the study following a two hour information session explaining the project, and following the completion of a signed consent form.

### Statistical Analysis

Grade outcomes for CCS assessment were scaled (A, B, C or D) non-parametric variables. Testing for differences was performed using the Mann-Whitney U test. The p value was ≤ 0.05.

## Results

Eight volunteers applied for the SPE training programme. Following interview by the UK SPE'e, 6 volunteers were enrolled and completed the two day training course. Five volunteers were considered of sufficient calibre to play consistent realistic roles, and to provide formative feedback to the students upon completion of the scenario. The sixth volunteer demonstrated good role-playing ability but was not comfortable in giving student feedback and therefore voluntarily withdrew from the project.

All eight academic participants completed the training workshop and were deemed capable of assessing student performance in CCS by the UK trainers.

**Figure 1:**
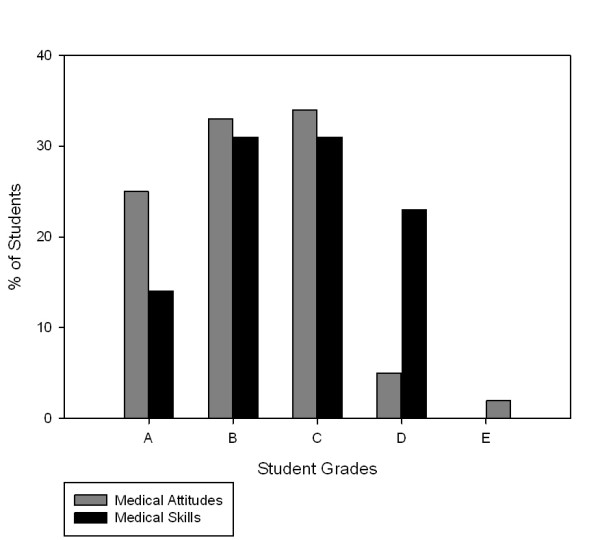
**Medical Student Grades Attitudes and Skills (n = 46)**.

**Figure 2:**
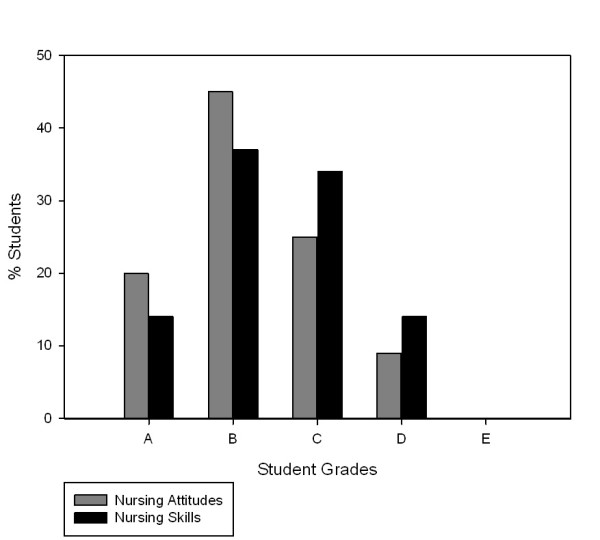
**Nursing Student Grades Attitudes and Skills (n = 46)**.

**Figure 3:**
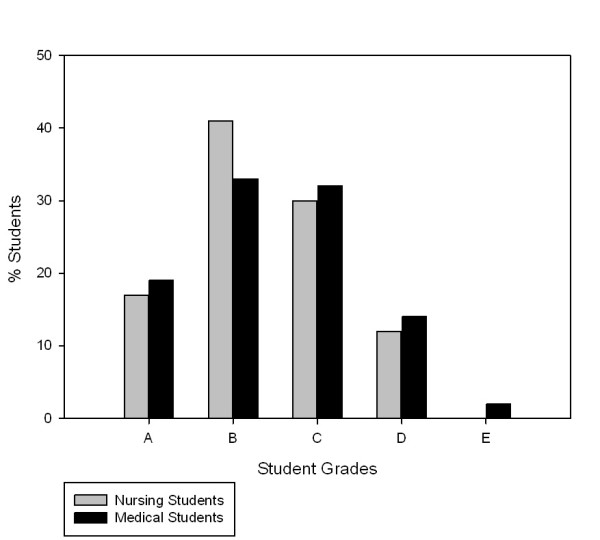
**Combined Medical and Nursing Grades Attitudes and Skills (n = 46)**.

Exactly 100 students (Nursing 64: Medicine 46) participated in the study. Although not statistically significant, there was a trend towards more nursing students (81%) passing both the communication skills and attitudes skills, compared to medical students (81% versus 75%; p = 0.055; table 1). There were no significant differences between the negotiated grades (academic rater and SPE) when comparing nursing and medical students' attitudes (p = 0.081) and communication skills (p = 0.698). Neither were there any significant differences between nursing and medical students' attitudes (p = 0.991) and skills (p = 0.125) when using academic ratings alone. Medical students and nursing student's negotiated grades (attitudes and skills) and combined grades, by the negotiated grade, are presented in the Figures 1, 2 and 3.

**Table 1:** Medical student and nursing student performance in CCS

Grade	Nursing students (N = 64)	Medical Students (N = 46)
Passed	52 (81%)	33 (72%)
Failed and Referred	12 (19%)	13 (28%)
Returned	2	9

All nursing students had English as their first language. Nine of thirteen medical students referred for enhanced learning in CCS did not have English as their first language. Nine of the 13 medical students who were referred for a one-on-one remedial CCS training session subsequently attended, compared to only 2 of the 12 referred nursing students.

## Discussion

Coherent communication can be described as *"authentic, non-judgmental, active listening for the essence, in order to understand the patient *[9]. It is said to occur when patients feel listened to, understood and acknowledged [10]. Good communication can also be described as *"the good man or woman (attitudes), talking well (skills)" *[11], with attitudes in this context referring to the student's professionalism. This project was driven by the knowledge that good CCS in clinical practice may improve patient health outcomes [12,13]. We were also aware that many students arrived at the clinical arena in the workplace, without their CCS being formally assessed at any time during their training. We had no prior concept of what proportion of students would pass and what proportion would pass/fail a validated SPE based scenario in CCS.

This study showed that while the majority of students demonstrated good to excellent CCS, a significant proportion (20-25%) demonstrated the need for further remedial training in both the skills and the attitudes necessary for good communication. The failure rate in CCS assessment in our study was higher than that reported by Ross and colleagues [14]. In a study of 129 medical students, 9% (12/129) failed in at least one of the four OSCE communication scenarios while a significantly larger proportion displayed only average ability. This group suggested that offering remedial action (treating symptoms) may be less important than attempting to discover why students fail in the first place (mapping the causes).

Medical students had some obvious disadvantages compared to nursing students. Although all international medical students have to pass an internationally recognised English language assessment prior to acceptance into our medical school, the absence of English as a first language was predictably a significant issue for many. In addition, the nursing students (all native English speakers) were more advanced in their studies compared to medical students. Indeed, if students who did not have English as a first language (who accounted for 9/13 medical student remediation referrals) were excluded, medical students had a better pass rate. That the majority of medical students referred for remedial training did not have English a first language has been shown to be a significant factor in poor CCS [15]. Fernandez and colleagues examined the impact of student ethnicity and primary childhood language on the communication scores in medical students. Even after controlling for English language knowledge, speaking a primary childhood language other than English was associated with lower communication scores for Asian students. Thus, caution is indicated when interpreting communication scores among culturally diverse students. Similarly, we have to be careful about the predictive ability of a single assessment of CCS. Language proficiency and cultural diversity would not explain why one in five nursing students did not perform well in our study. There may be other reasons for their underperformance. A fundamental educational principle is that there should be an alignment between instruments of learning and instruments of assessment [16]. This is particularly important when assessing CCS. This is a limitation of our study, since we *assessed *by SPE scenario testing, but the students were *not taught *in this manner. In fact, this project was the students' first exposure to SPE's. In addition, the death of a peer during the assessment period may have understandably impaired nursing student performance and subsequent attendance at the remedial sessions.

There is an ongoing debate between a holistic approach to teaching and assessing CCS using global rating scales such as those used in this study, and the checklist of observable competencies such as the Calgary-Cambridge system [15]. Without doubt there are advantages and disadvantages to both. One of the criticisms of the global approach is that it may set the students up to fail by not providing a structure or competencies that they can acquire in a systematic manner. Equally, one might argue that a global approach is impressionistic and may not be reliable. The counter arguments are that patients do not break communication down into competencies, and that the negotiation of meaning, (which is after all what communication is) occurs across different levels from sensor to receiver. In addition, it cannot be assumed that every receiver will react in the same way to each competency. The holistic view is about developing the student's ability to reflect and adapt their communication styles to different individuals. The holistic, negotiated evaluation tool used in this study was developed and validated by our collaborators at the University of Birmingham, UK. Wiskin and colleagues [8] showed that a negotiated mark between assessor and SPE fulfils the psychometric requirements of validity, consistency and accuracy, in addition to being feasible in terms of cost and time. We found no significant differences between assessor and SPE grading of both attitudes and skills in the current study. While the newly trained Irish assessors were indeed novice raters, they were all experienced medical and nursing educators, most of whom were directly involved daily in teaching of CCS. In addition, at every scenario, the assessment duo consisted of a newly trained Irish rater (academic or SPE) matched to experienced UK rater. Wiskins et al [8] has shown that the input into examiner training, not examiner experience, was the more important variable in using the VOICE rating scale. They demonstrated consistency over 4 years, using a global holistic scale, with examiners of mixed previous experience.

Student ratings were based on a random choice from five role play scenarios. Whether students might have done better on the scenarios they were not tested upon (as with any examination) is speculative and cannot be determined from these data. Assessing a CCS scenario is complex and fraught with confounding variables including the patient (SPE), the examiner, and student knowledge of the clinical content, the clinical task, and the context. Annie Cushing, in an in-depth review of these variables, concluded that "Reliable assessment of an individual's communication skills using any one assessment tool is problematic" [17]. However, she added that "there is a move towards global rating scales for summative assessment. Checklists are useful for specifying behaviours in learning and feedback contexts, while global instruments would seem to fulfil the requirements of reliability, validity and provide easier more practical and feasible methods". In our study we tried to control for the content variability by using a small number of scenarios and downplaying the need for student knowledge of content prior to the scenario and the assessment.

Most medical students referred for remedial training attended while most nursing students did not. Since the majority of these medical students did not have English as a first language, they may have had greater insight into their level of CCS and are receptive to additional training. We did not have a protocol to deal with students who were referred but did not attend the remedial session (the majority of whom were nursing students). For some students, failure in the critical arena of CCS may have affected their self-belief and confidence. Therefore, referred students need not only remedial help but also psychological support. It is appropriate that these students receive further advice and training from independent experts in CCS, along with an early opportunity to demonstrate competency. It has been the experience of our collaborators from the University of Birmingham that remedial advice may not necessarily be beneficial coming from the student's clinical teachers.

There is a concern that mastery of skill in a laboratory or in theory does not necessarily mean that students will be able to utilize those skills in clinical practice. Chessman and colleagues showed that good CCS may not in fact be predictive of future performance. They showed no relationship (R = 0.08) between students CCS performance in a laboratory and subsequent performance in clinical examinations [18], a finding that has been supported by others [19,20]. Thus, the need to integrate communication skills training with practice learning is a recurrent theme in medical education and implies that communication skills should be taught and assessed at undergraduate, post graduate and clinical practice levels. Since one of the purposes of evaluation of student performance is to assure future good quality clinical performance, then the variety of challenges in communication that will confront the professional in real life should be included. As Paulo Freire, the great Brazilian educator, (who conceptualized education as a means to empower street children with ways of understanding their world and changing it), said: *"Teaching demands an understanding that education is a form of intervening in the world."*

What is the evidence for using SPE's for assessing CCS? Research supports reliability of portrayal and data capture by SPE's, as well as the predictability of future trainee performance [21]. SPE's are considered to represent a potentially more objective means for assessment, particularly in the area of CCS [22], and are used by over 80% of North American medical schools and Licensure Examinations to assess competencies [23,24].

This study raises the question whether students who fail to demonstrate a minimal level of competence in this area should to be allowed to progress to the next stage of the course and eventually graduate. Dowell and colleagues used 'barrier' stations in communication skills in their OSCE. Students who failed these stations, irrespective of their overall exam performance, had to undertake a compulsory 2-week module in CCS followed by assessment before being allowed to progress [25].

Are the results of this study generalizable to other Medical and Nursing schools with similar student profiles? We think so. The scenarios we tested on were realistic and reflected the range of problems students would expect to be exposed to during clinical placements in the hospital or the community settings. Other national educational institutions are facing similar issues to us [26]. They may welcome collaboration in furthering the results of these studies. In addition, regulatory/licensing bodies may be willing to provide support for such collaboration, as they have the ultimate responsibility to ensure CCS competency in Health care graduates.

Why did we choose to assess and compare nursing and medical students together in this project? The nursing and medical students were not directly comparable in terms of training and teaching programmes. However, both groups had just completed their formal CCS training. The scenarios used in our study were the same narrative for both groups but students were expected to act within and according to the scope of their own discipline-specific roles. Health Care communication skills are generic and are subsequently used in the same work-based contexts by both future nurses and doctors. Indeed there is a good case for health care professionals to be taught and assessed in their CCS, as an interdisciplinary team, not uni-professionally as occurs generally [27].

This study was an international interprofessional collaborative project between multidisciplinary teaching staff. The researchers, faculty staff, lay role players, students, along with our international collaborators were all learning in action, or learning experientially. The project challenged competencies in the knowledge, skills and attitudes of CCS not only of students but also of the academic assessors. The interprofessional benefit for the students was seeing their teachers collaborating across disciplines in communication skills, a common, but critical area of education and practice. Students also experienced assessment by non-disciplinary colleagues and by lay SPE's. If the contact theory proves correct, all participants may have gained an understanding of the challenges facing their fellow professionals and dispelled some myths and stereotypes. The Contact hypothesis [28] suggests that friction between different social groups can be alleviated if they can interact with one another, provided certain conditions are met. These include equality of status, the need for members to work on common goals in a cooperative manner, and the need for members to focus on understanding differences and similarities between themselves.

## Conclusions

We conclude with the following recommendations for schools interested in assessing CCS in undergraduate health care education. In order for assessments in CCS to be reliable and valid, ideally there should be an alignment between the mode of teaching and modes of assessments, i.e. students being evaluated by SPE's, should expect to learn their CCS through SPE's. A single assessment of CCS during the Schools curricula is probably insufficient. Testing should occur at a minimum of two occasions during undergraduate training. Students who are not deemed competent in communication skills should be offered remedial training, preferably by professionals outside their academic department. The language and/or cultural challenges in CCS, faced by international students without English as a first language, needs to be urgently addressed. Finally, schools need to develop policies and procedures to address students who persistently fail CCS assessments, including career redirection, if appropriate.

## Competing interests

The authors declare that they have no competing interests.

## Authors' contributions

CAR, NW and RG designed and implemented the study. AS and CW contributed to the design and participated in the training and assessment components. LB was involved in the data gathering and analysis and helped to draft the manuscript. All authors read and approved the final manuscript.

## Pre-publication history

The pre-publication history for this paper can be accessed here:

http://www.biomedcentral.com/1472-6920/10/24/prepub
